# Infantile primary carnitine deficiency: A severe cardiac presentation unresponsive to carnitine supplementation

**DOI:** 10.1002/jmd2.12346

**Published:** 2022-11-09

**Authors:** Lebreton Louis, Gaschignard Margaux, Guibet Claire, Lamireau Delphine, Roche Sandrine, Richard Emmanuel, Ged Cécile, Mesli Samir, Redonnet‐Vernhet Isabelle

**Affiliations:** ^1^ Laboratoire de Biochimie Pôle de Biologie et Pathologie, CHU de Bordeaux Bordeaux France; ^2^ Hôpital Pédiatrique, Pôle Pédiatrique, CHU de Bordeaux Bordeaux France; ^3^ INSERM BRIC U1312 Université de Bordeaux Bordeaux France; ^4^ lNSERM MRGM U1211 Université de Bordeaux Bordeaux France

**Keywords:** cardiomyopathy, children, fatty oxidation, primary carnitine deficiency

## Abstract

Primary carnitine deficiency (PCD) is an inherited disease of fatty acid beta‐oxidation with autosomal recessive inheritance. The disease manifests as metabolic decompensation with hypoketotic hypoglycaemia associated with cardiomyopathy, hepatomegaly, rhabdomyolysis, and seizures. Various outcomes are described from asymptomatic adults to dramatic sudden infant death syndrome cases. We present a severe case of PCD decompensation in an 18‐week‐old female. She presented with hypotonia, moaning, diarrhea, and vomiting at the pediatric emergency. Initially suspected as intracranial hypertension, the clinical condition evolved rapidly and caused a reversible cardiac arrest with profound hypoglycemia. Despite carnitine supplementation, she succumbed from cardiac arrhythmia and multivisceral failure 4 days after admission. The genetic analyses showed a PCD with biallelic pathogenic variants of *SLC22A5* gene. The case report is notable for the severity of the cardiac damage possibly favored by maternal carnitine deficiency during pregnancy. The analysis of previously published PCD cases highlights (i) the importance of having large access to emergency biochemical tests for early therapeutic care although the disease has unpredictable severity and (ii) the fact that the clinical outcome remains unpredictable if carnitine treatment is initiated late.

## 1 INTRODUCTION

Primary carnitine deficiency (PCD) is an autosomal recessive metabolic‐inherited disease of the carnitine cycle (OMIM no. 212140) caused by a carnitine transporter OCTN2 (organic cation transporter protein)^1^ defect that results in defective fatty acid oxidation. The incidence of PCD varies according to populations as shown by neonatal screening programs with a frequency of ~1/20 000–1/70 000 in Europe,^2^ 1/40 000 in Japan,^3^ 1/140 000 in United States,^4^ and 1/300 in Faroe Islands^5^ due to a founder effect.

Localized in the cytoplasmic membrane, OCTN2 is encoded by the *SLC22A5* gene (solute carrier family 22 member 5) located on chromosome 5q31.1. Carnitine is an essential water‐soluble molecule for the transfer of long‐chain fatty acids across the inner mitochondrial membrane with subsequent β‐oxidation. Defects in the OCTN2 carnitine transporter result in urinary carnitine wasting, low serum carnitine levels, and intracellular carnitine deficiency. Since their supplied energy mainly comes from fatty acids beta‐oxidation, myocardium and skeletal muscles are particularly sensitive to carnitine deficiency.

Clinical manifestations of PCD vary widely from infant sudden death to asymptomatic adult forms.^6^ Between 3 months and 2 years of age the metabolic presentation is more frequent^7^ with hypoketotic hypoglycemic episodes, hepatic encephalopathy, lethargy, hyperammonemia, hepatomegaly, and elevated transaminases. Seizures, triggered by fasting or febrile episodes, can leave neurological sequelae that will result in mental retardation or learning difficulties. Lack of treatment can lead to cerebral edema, seizures, coma, and death. Patients with PCD can develop dilated cardiomyopathy, usually between 1 and 7 years of age, sometimes associated with proximal myopathy giving rise to muscle weakness and amyotrophy^7^, ^8^, ^9^, ^10^ Adult patients with PCD are in most cases asymtomatic^11^ or present with minor symptoms as muscle weakness. However, adults are at risk of sudden death from cardiac arrhythmia.^12^, ^13^, ^14^, ^15^, ^16^, ^17^ Asymptomatic mothers with PCD were identified through their baby's newborn screen by extremely low free carnitine levels reflecting the mother's condition.^15^, ^18^, ^19^ PCD outcome partly depends on early diagnosis and carnitine supplementation.^6^

Herein, we present the case of an 18‐week‐old female child diagnosed with PCD during a severe and fatal episode of metabolic and cardiac decompensation.

## 2 CASE REPORT

### 2.1 Clinical and biochemical presentation

An 18 week‐old female child was admitted at the pediatric emergency of Bordeaux University Hospital presenting hypotonia, moaning, diarrhea and vomiting. Second child from nonconsanguineous parents, she was born at term with normal weight and she has presented since birth many unexplained crying episodes that became more frequent in the last weeks. She had weight gain difficulties from the first weeks of life in the context of exclusive breastfeeding then switched to artificial milk. The progressive occurrence of diarrhea and vomiting with lethargic episodes led the parents to bring their child to the pediatric emergency department.

On admission to hospital, hemodynamics showed a heart rate of 140 bpm interspersed with episodes of bradycardia and arterial hypertension, deep respiration (respiratory rate = 40) with normal oxygen saturation. Hepatomegaly was found by abdominal palpation of a 3 cm liver span. Neurological exam showed Glasgow scores ranging from 11 to 15, fluctuating consciousness, generalized hypotonia, no signs of focusing, no apparent motor deficiency, and normotensive fontanel. She had no fever.

Mannitol administration was quickly started (1 g/kg) to prevent intracranial hypertension. In addition, antibiotic prophylaxis (Ceftriaxone and Gentamicin) and hydratation (NaCl 0.9%) were set up.

No abnormalities were identified on brain computed tomography scan and on thoracic x‐ray.

Biochemical testing (Table 1) highlighted metabolic acidosis with hyperlactatemia, hepatic cytolysis, hyperammonemia, cardiac, and hepatic functions. Hematology and other common parameters were in the normal range, no infectious agent was detected, blood glucose was not tested at admission.

**TABLE 1:** Laboratory testing at entrance

Parameter	Value	Normal range
pH	7.27	7.32–7.43
Bicarbonate	15.1 mmol/L	23–26 mmol/L
Lactate	2.5 mmol/L	0.5–1.5 mmol/L
Troponin	349 ng/L	<16 ng/L
AST	997 UI/L	5–34 UI/L
ALT	469 UI/L	6–55 UI/L
Ammonia (venous)	227 μmol/L	18–72 μmol/L
Creatine kinase	616 UI/L	29–168 UI/L
Urea	8.6 mmol/L	1.2–6.0 mmol/L

Abbreviations: ALT, alanine transaminase; AST, aspartate transaminase.

Thus, 3 h after admission she was transferred to an intensive care unit. On arrival, the child had seizures with generalized hypertonia and was recovered from cardiorespiratory arrest through an intensive care management. At that time, capillary blood glucose level was 0.18 g/L, treated by 10% glucose infusion at the dose of 8 mg glucose/kg/d.

Immediately after recovering from cardiorespiratory arrest a bolus of carnitine (200 mg/kg) and a bolus of sodium benzoate (250 mg/kg/4 h) were administered.

The biological follow‐up showed a majoration of hyperammonemia (354 μmol/L) further treated, after metabolic advice, by carglumic acid (Carbaglu®; 50 mg/kg/6 h) and a second bolus of sodium benzoate (200 mg/kg). The ammonia level dropped to 121 μmol/L 3 h after that treatment and to 72 μmol/L after 5 h. Additionally, an exclusive carbohydrate diet was started and a treatment with carnitine at 50 mg/kg/d was instaured.

Left ventricular enlargement as well as severe cardiac failure (ejection fraction 25%–30%) were shown by cardiac ultrasound, requiring transfer to a specialized cardiac care unit 1 day after admission.

Results from the inherited metabolic diseases department were available 3 days after admission. First, an elevated medium and long‐chain dicarboxylic aciduria was found in the chromatography of urinary organic acids (OA), in good agreement with a long chain fatty acids beta‐oxidation defect. Then the deeply decreased free blood carnitine (0.5 μmol/L; normal range: 33–44 μmol/L) and total blood carnitine (1.36 μmol/L; normal range: 59–80 μmol/L) levels raised the suspicion of PCD. Consequently carnitine treatment was increased from 50 to 200 mg/kg/d at Day 3.

In summary, the doses of carnitine injected were a bolus of 200 mg/kg after cardiac recovery (Day 1), then 50 mg/kg (Days 1 and 2), and 200 mg/kg (Day 3 and 4).

Unfortunately, despite high doses of carnitine, she presented severe episodes of arrhythmia with long QRS complex and supraventricular tachycardia (200/300 bpm), an oedematous syndrome and finally a multisystemic failure leading to death 4 days after hospital admission.

### 2.2 Genetic analysis and family testing

The diagnosis of PCD was further confirmed by *SLC22A5* gene analysis (reference transcript NM_003060.4). Two heterozygous variants were found and classified as pathogenic, an already described^20^ maternally inherited missense c.839C > T (p.Ser280Phe) variant, and a paternally inherited nonsense c.1006C > T (p.Arg336*) variant.

In contrast to the patient, carnitine testing and molecular study performed in the parents and the 3‐year‐old brother showed low levels of free and total carnitine in good agreement with an herozygous status (Table 2).

**TABLE 2:** Familial analysis: genetic outcome and carnitine levels

	PCD signs	Plasma carnitine		SLC22A5 (NM_003060.4) variants	
		Free (NR)	Total (NR)	c.839C > T p.Ser280Phe	c.1006C > T p.Arg336*
Patient	Yes	0.5 (33–43)	1.36 (59–80)	Heterozygous	Heterozygous
Father	No	14 (31–37)	23 (54–67)	Absent	Heterozygous
Mother	No	9 (31–37)	15 (54–67)	Heterozygous	Absent
Elder brother	No	6 (34–39)	12 (57–70)	Absent	Heterozygous

Abbreviation: NR: normal range (μmol/L).

## 3 MATERIALS AND METHODS

### 3.1 Carnitine and acylcarnitine analysis

Acylcarnitines, total and free carnitine quantifications were performed by electrospray tandem mass spectrometry (Waters Quattro micro™) from dried blood spots using the nonderivatized kit MassChrom® Amino Acids and Acylcarnitines (Chromsystems).

### 3.2 Urine OA profile

Urine OA detection and quantification were performed by gas chromatography–mass spectrometry Clarus 500™ (Perkin Elmer) after liquid/liquid extraction in ethyl acetate and derivatization by (*N*,*O*‐Bis[trimethylsilyl]trifluoroacetamide).

### 3.3 Genetic analyses

Genomic DNA was extracted from peripheral blood by automated method (TECAN freedom EVO). The *SLC22A5* exons and flanking regions (±25 bp) were amplified using a polymerase chain reaction‐based method designed through Ampliseq designer (Thermofisher). The library was prepared using Ion AmpliSeq Library kit 2.0‐96LV and Ion CHEF (Ion Torrent by Thermo Fisher Scientific); the sequencing was performed with Ion S5XL (Ion Torrent by Thermo Fisher Scientific); the bioinformatic worflow utilized TorrentSuite for base calling, alignment and variant calling steps; the biological interpretation was conducted following the ACMG classification. The Ampliseq design also contained other genes to ruled out other betaoxydation defects: *ACADVL, CPT1A, CPT2, ETFA, ETFB, SLC25A20, HADHA, HADHB, ACAD9, ACADS,* and *ACADM*.

Sanger sequencing was performed for relatives with targeted analysis of the Exons 5 and 6 of *SLC22A5* (NM_003060.4) gene, using an 3500xl genetic analyzer (Applied Biosystems).

## 4 DISCUSSION

We reported a severe case of PCD decompensation in an 18‐week‐old female infant. Initially, a few days before admission, the infant presented gastrointestinal disorders. First, metabolic evaluation evidenced cardiac, renal and hepatic involvement, metabolic acidosis, and hyperammonemia. The condition worsened rapidly after admission with seizures followed by cardiorespiratory arrest. After recovery, dilated cardiomyopathy with left ventricular insufficiency was evidenced which rapidly caused malignant arrhythmia resistant to drug treatment and cardioversion. Despite implementation of high doses of oral carnitine, irreversible multivisceral failure led to the decision to discontinue supportive care.

According to the review of Shibbani et al.,^9^ cardiac involvement is very common in the PCD patients. Nevertheless, cardiac presentations are usually described after 1 year of life and hepatic presentations are the most frequent before 2 years of age. Thus, to our knowledge, only nine infant PCD cases presenting with cardiac involvement before 1 year of age were published (Table 3). These patients had signs of respiratory infection or metabolic decompensation and showed evidence of altered cardiac function during evaluation. For almost all of them, cardiac function was totally or partially recovered by carnitine therapy within a few months and further metabolic decompensation was prevented. Tang et al.^21^ described the case of PCD twins who died at 6 months of life in a context of acute metabolic decompensation complicated by cardiac arrhythmias but in this particular case carnitine therapy was not provided.

**TABLE 3:** Review of infantile cardiac PCD cases reports (age of onset <1 year)

Age of onset	Clinical history before diagnosis	Plasmatic Free carnitine (NR: 33–43 μmol/L)	Carnitine supplementation	Cardiac function			Reference
				Initial	Recovery	Last follow‐up	
8 months	Respiratory infection	10.7	50 mg/kg/d	EF = 34%, LVE	EF 62%	4 months	Shibbani et al.^9^ (“Patient II‐2”)
11 months	Cough and poor feeding	8.8	50 mg/kg/d	EF = 28%, LVE	EF 56%	12 months	Shibbani et al.^9^ (“Patient VI‐4”)
11 months	None	10.3	Unknown	EF = 47%, LVE	EF 65%	12 months	Shibbani et al.^9^ (“Patient IV‐5”)
6 months	Encephalopathy, seizures	0.72	150 mg/kg/d (first year) 100 mg/kg/d (then)	LVE	Full recovery	17 months	Lamhonwah et al.^23^
7 months	Breathing shortness, congestive heart failure	1	Intravenous and per os 100 mg/kg/d	LVE	Full recovery	11 months	Rahbeeni et al.^30^
6 months	Breathing shortness	low	Unknown	Cardiac fibroelastosis	Full recovery	Unknown	Di san Filippo et al.^22^
5 months	Metabolic deterioration following gastroenteritis	<1	Unknown	unknown	Full recovery	Unknown	Di san Filippo et al.^22^
6 months	Acute metabolic derangement	9	No	unknown	Fatal demise		Tang et al.^21^
3 months	Episodes of cardiac arrest with severe hypoglycemia	low^a^	variable dosing^b^	LVE	Full recovery	20 years	Cederbaum et al.^31^ (“Case 1”)

Abbreviations: EF, ejection fraction; LVE, left ventricle enlargement; NR, normal range; PCD, primary carnitine deficiency.

^a^ In vitro testing.

^b^ Titrated to maintain plasma carnitine in the low to mid normal range.

Thus our case report is notable both for the severity of the cardiac damage by 18 weeks of age and for unresponsiveness to high doses of carnitine.

Could the genotype, in association with other intrinsic or environmental factors, explain such a severe cardiac presentation in our patient?

This case had a compound heterozygous status combining a nonsense and a missense variant classified as pathogenic. The nonsense variant causes a loss of function and the missense variant p.S280F, located in cytoplasmic domain, was shown to alter cations transport by OCTN2 in CHO cells.^22^ Both variants are rare in the gnomAD population and phenotype–genotype correlations are not available for these variants. Biallelic nonsense variants are more present in symptomatic than in asymptomatic patients^20^ and are associated with lower free carnitine levels^9^ than biallelic missense and mono‐allelic nonsense variants. No clear relationship between severity and predicted functional consequences of missense variant was found.^9^, ^23^ There is no correlation between genotype and cardiac or metabolic phenotype in PCD since different types of presentation have been observed within an individual family.^20^, ^23^, ^24^, ^25^ Moreover, we cannot exclude other factors, whether genetic, including nonexplored genes, or environmental, which may influence cardiac response to the lack of energetic supply caused by PCD. Such severe cardiac presentation may be explained by pre‐existing cardiac susceptibility that could be explored by large‐scale NGS sequencing. Thus, we did not find any other pathogenetic variant in the analyzed beta‐oxydation genes (cf Section 3).

In addition, analyses revealed no infectious diseases, and particularly no gastrointestinal infection that could promote a catabolic state and favor metabolic decompensation. Table 3 shows that some case reports had clinical manifestations of infectious diseases at the time of PCD disclosure.

In our case, the patient's heterozygous mother had very low levels of free and total carnitine in agreement with a secondary carnitine deficiency (Table 2). This could have negatively impacted the child's phenotype, by causing low tissue carnitine stores at birth. Normal or borderline low levels of plasma carnitine are described in heterozygous parents of affected children^23^, ^26^ but most of the publications do not report parental and siblings carnitine levels. In heterozygotes for PCD, a low protein dietary intake (vegetarian diet) in association with a less effective renal carnitine reabsorption^26^ could result in low levels of plasma and tissue carnitine, as exogenous carnitine constitutes about 75% of the carnitine pool.

The index case's older brother had also very low levels of free carnitine deficiency (6 μmol/L) the difference >5 μmol/L between free and total carnitine was consistent with heterozygous status.^27^ Echocardiography and clinical examination at 2‐year‐old were normal. Genetic analysis confirmed the heterozygous carrier status. Only Exons 5 and 6 of SLC22A5 genes were analyzed for the relatives.

The occurrence in our case of sudden cardiac signs moving towards a rapidly fatal cardiovascular decompensation although high doses of carnitine treatment, and comparative literature show both that (i) under 1 year of age cardiovascular damages in PCD are very frequent and most of the time at subclinical level (ii) clinical outcome remains unpredictable if carnitine treatment is initiated late. These data reinforce the interest of neonatal screening in PCD, despite a low predictive positive value^28^ and the decision to discontinue screening in some countries.^29^ Neonatal screening in PCD would allow newborns to be immediately treated with L‐carnitine in order to avoid future fatal complications.

## 5 CONCLUSION

The PCD case reported had an early fatal demise following metabolic and cardiac decompensation. The absence of striking clinical signs before admission delayed the appropriate management of the patient. Factors influencing the severity of the phenotype were not clearly identified. A secondary carnitine defect in the mother due to the association of poor diet and decreased renal reabsorption could be a part of the explanation. In case of new pregnancy, carnitine supplementation should be provided to the mother until the genetic testing results in foetus are obtain. The unresponsiveness to carnitine therapy in this severe case highlights the importance of expanding neonatal screening to PCD.

## AUTHOR CONTRIBUTIONS

Lebreton Louis: analysis and interpretation of data + drafting the article. Gaschignard Margaux: analysis and interpretation of data. Guibet Claire: analysis and interpretation of data (bibliographic research). Lamireau Delphine: revising the article critically for important intellectual content. Roche Sandrine: revising the article critically for important intellectual content. Richard Emmanuel: head of biochemistry department at Bordeaux University Hospital. Ged Cécile: revising the article critically for important intellectual content. Mesli Samir: revising the article critically for important intellectual content. Redonnet‐Vernhet Isabelle: analysis and interpretation of data + revising the article critically for important intellectual content.

## CONFLICT OF INTEREST

The author declares that there is no conflict of interest that could be perceived as prejudicing the impartiality of the research reported.

## INFORMED CONSENT

All procedures followed were in accordance with the ethical standards of the responsible committee on human experimentation (institutional and national) and with the Helsinki Declaration of 1975, as revised in 2000 (5). Informed consent was obtained from all patients for being included in the study.Proof that informed consent was obtained must be available upon request.

## ANIMAL RIGHTS

No animal in that study.
